# Developing a healthcare dataset information resource (DIR) based on Semantic Web

**DOI:** 10.1186/s12920-018-0411-5

**Published:** 2018-11-20

**Authors:** Jingyi Shi, Mingna Zheng, Lixia Yao, Yaorong Ge

**Affiliations:** 1 Department of Software and Information Systems, University of North Carolina at Charlotte, 9201 University City Blvd, Charlotte, 28223 NC USA; 2 Department of Health Sciences Research, Mayo Clinic, 200 First Street SW, Rochester, 55905 MN USA

**Keywords:** Health informatics, Dataset information resource, Knowledge representation, Semantic web, Knowledge extraction

## Abstract

**Background:**

The right dataset is essential to obtain the right insights in data science; therefore, it is important for data scientists to have a good understanding of the availability of relevant datasets as well as the content, structure, and existing analyses of these datasets. While a number of efforts are underway to integrate the large amount and variety of datasets, the lack of an information resource that focuses on specific needs of target users of datasets has existed as a problem for years. To address this gap, we have developed a Dataset Information Resource (DIR), using a user-oriented approach, which gathers relevant dataset knowledge for specific user types. In the present version, we specifically address the challenges of entry-level data scientists in learning to identify, understand, and analyze major datasets in healthcare. We emphasize that the DIR does not contain actual data from the datasets but aims to provide comprehensive knowledge about the datasets and their analyses.

**Methods:**

The DIR leverages Semantic Web technologies and the W3C Dataset Description Profile as the standard for knowledge integration and representation. To extract tailored knowledge for target users, we have developed methods for manual extractions from dataset documentations as well as semi-automatic extractions from related publications, using natural language processing (NLP)-based approaches. A semantic query component is available for knowledge retrieval, and a parameterized question-answering functionality is provided to facilitate the ease of search.

**Results:**

The DIR prototype is composed of four major components—dataset metadata and related knowledge, search modules, question answering for frequently-asked questions, and blogs. The current implementation includes information on 12 commonly used large and complex healthcare datasets. The initial usage evaluation based on health informatics novices indicates that the DIR is helpful and beginner-friendly.

**Conclusions:**

We have developed a novel user-oriented DIR that provides dataset knowledge specialized for target user groups. Knowledge about datasets is effectively represented in the Semantic Web. At this initial stage, the DIR has already been able to provide sophisticated and relevant knowledge of 12 datasets to help entry health informacians learn healthcare data analysis using suitable datasets. Further development of both content and function levels is underway.

## Background

Healthcare data is rapidly growing in the era of big data. An increasing number of researchers are leveraging these datasets to improve the quality of patient care. However, challenges caused by a variety of purposes, designs, and techniques when health data were originally collected boost the complexity and diversity of healthcare datasets. For health data analysis, it requires significant time, energy, and fundamental knowledge to identify, understand, and choose the right datasets. The challenges for students and researchers who have little experience are even more pronounced. A number of online data resources, such as HealthData.gov [1], Data.CDC.gov [2], and Society of General Internal Medicine (SGIM) Research Dataset Compendium [3], integrate basic information for public datasets, which help new investigators choose datasets to a certain extent. However, the simple descriptions in these portals are hardly adequate for them to identify a suitable dataset to delve into. Simple search functions, such as a keywords search, provided by most of the resources cannot handle more complex and less concrete questions that typical novices have, such as finding existing analytical methods that are suitable for analyzing a particular dataset. Meanwhile, proprietary datasets, often having limited information in these portals, are even harder to understand and analyze.

Noticing these shortcomings, emerging research projects are attempting to build structured dataset information resources that address the challenge of dataset discovery and accessibility. For example, the Stanford University School of Medicine established the Center for Expanded Data Annotation and Retrieval (CEDAR) project in 2015 to facilitate researchers’ standard use of metadata by developing an authoring-friendly computational ecosystem for metadata development, evaluation, use, and refinement [4]. By 2017, they had developed a CEDAR Workbench, which was an ontology-assisted tool to help scientific experiment metadata authoring [5]. Meanwhile, the University of California at San Diego is leading the development of a data discovery index system, the biomedical and healthCAre Data Discovery Index Ecosystem (bioCADDIE) [6], to index data that are stored elsewhere to facilitate data integration tasks that adopt content standards and high-level schema. A prototype biomedical data search engine, DataMed [7], under the bioCADDIE project, has included metadata extracted from multiple biomedical data repositories, such as the Cambridge Crystallographic Data Centre (CCDC) and U.S. National Center for Biotechnology Information (NCBI)’s BioProject. Similar to what PubMed (a free search engine that comprises more than 28 million citations from multiple literature databases and resources) has done for the biomedical literature, DataMed aims to make a comparable contribution for biomedical data.

However, the current attempts, focusing on integrating and searching datasets and dataset information, often lack consideration of the learning needs of specific target user populations. Particularly, there is no resource specifically designed to address the needs of health informatics students and novice researchers. Their learning curve is considerably steep when they explore datasets using existing resources. We believe the lack of a healthcare dataset information resource that brings information from various resources together to address the unique needs and questions from these learners is an important gap in health informatics development.

To bridge the gap, we have developed the Dataset Information Resource (DIR) framework, specifically aimed at helping entry-level health informatics students and researchers. For these novices, the challenges are different from established researchers. It is not the discovery of datasets that is important. Rather, the importance lies in the surveying of the landscape of existing datasets and the identification of a proper dataset from the set of common datasets for a given problem. Additionally, the understanding of the dataset and related analytical methods is critically important. The DIR framework does not contain actual data from the datasets. Instead, it is a specialized knowledge base that provides comprehensive knowledge and answers sophisticated questions about noteworthy datasets that address the needs of beginning learners. Besides common information about datasets, such as descriptions, we focus more on knowledge needed by novices, such as analytical methods that datasets can utilize. In this case, novices can quickly obtain a solid understanding through concrete cases. Moreover, we provide dataset blogs in the DIR so that users can easily start data analysis by following sample codes and instructions.

For a flexible, meaningful, and robust knowledge representation, we leveraged Semantic Web [8] technologies. Meanwhile, we incorporated the W3C Dataset Description Profile standard [9] developed by the Semantic Web Health Care and Life Sciences (HCLS) interest group to ensure that the metadata delivered are well defined and organized. The current DIR prototype focuses on 12 representative datasets in healthcare, including both public and proprietary datasets. The prototype is published and accessible via https://cci-hit.uncc.edu/dir/.

## Methods

The DIR framework is based on Semantic Web technologies. Building on them, we developed methods to extract knowledge from the datasets as well as existing research articles that had analyzed these datasets. We also developed a question-answering module that answered novice questions that had been posted on the web. In the following sections, we briefly describe the Semantic Web first and then describe the system design, major components, knowledge representation and extraction, and dataset learning of the DIR framework.

### Semantic web

The Semantic Web is an extension that adds semantics and logic to the well-known World Wide Web (WWW). In the traditional web pages, entities, such as concepts, are dispersed in the text. They are not clearly identified and their relationships are not explicitly represented. In contrast to traditional web pages, the Semantic Web enhances the regular web by coding and linking important concepts. Therefore, it makes semantics behind data understandable not only to human beings but also to machines. The Semantic Web is based on the Resource Description Framework (RDF) [10]. To link entities, RDF provides a straightforward syntax for describing resources, which is called “triple”. An RDF triple contains three components—the subject, predicate, and the object, where the predicate represents the relationship between the subject and the object. To query the linked entities, a query language, SPARQL Protocol and RDF Query Language (SPARQL) [11], is designed, which is the key to reasoning. With the support of these techniques, a number of RDF-based resource frameworks have already been developed that show the power of the Semantic Web, such as DBpedia [12] and the Neuroscience Information Framework (NIF) [13].

### DIR framework overview

**Fig. 1:**
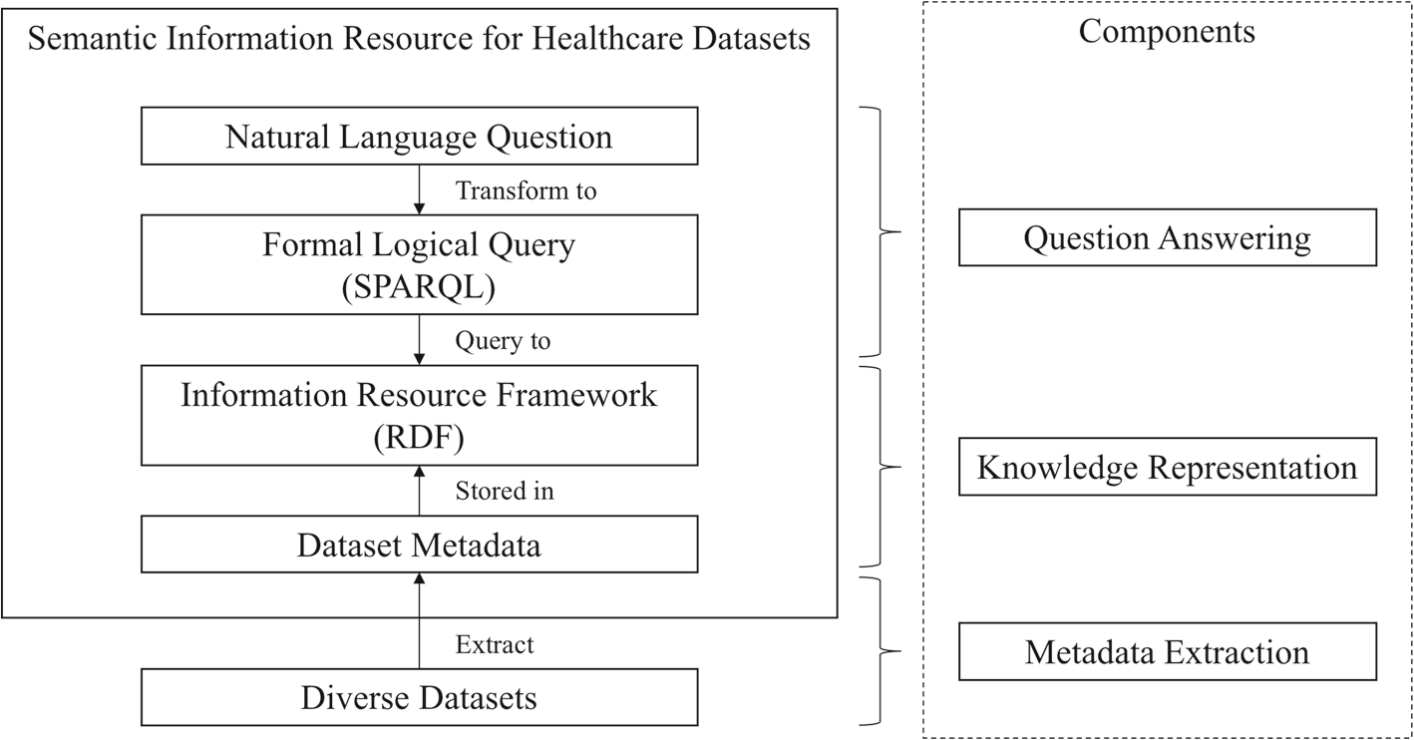
Proposed architecture of DIR system

The proposed architecture of the DIR system is shown in Fig. 1. It consists of three major components: (1) knowledge representation (requires the ability to represent metadata in a flexible, extendable, and reusable way to meet and surpass the FAIR Data Principles [14]), (2) question answering (delivers exact knowledge to novices), and (3) metadata extraction (extracts metadata tailored to novices from a large number of diverse dataset resources). With these components, the system can integrate and represent knowledge from scattered datasets, allow flexible research questions, and provide precise answers at a suitable level of comprehension.

**Fig. 2:**
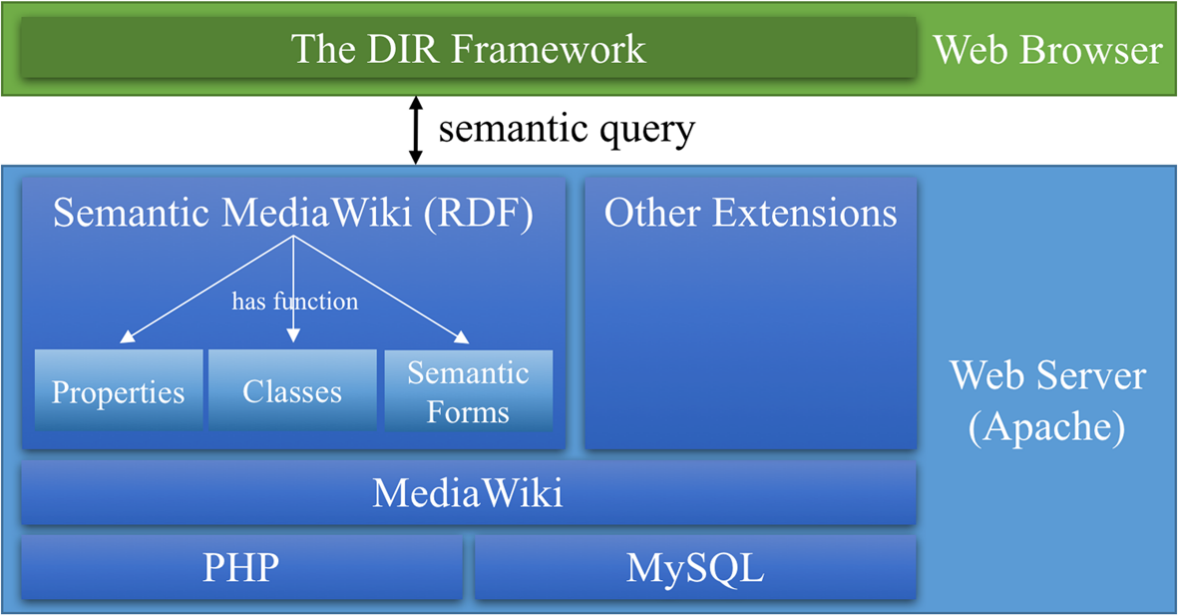
Infrastructure of DIR prototype

The DIR prototype is built on top of the open-sourced Semantic MediaWiki (SMW) platform [15] for knowledge representation and question answering. SMW, which tightly couples traditional web pages with an RDF representation to capture essential knowledge, is an extension of MediaWiki (MW) [16] (see Fig. 2). Additionally, MW is well-known as the foundation of Wikipedia, whose English site contains 5,605,853 articles. Therefore, advantages of MW—such as stability facing massive content and heavy traffic—and advantages of SMW—including the embedded functionality to represent RDF triples by using properties, classes, and semantic forms—can be fully leveraged. Once the knowledge of diverse datasets is extracted, SMW provides a platform for representation and a SPARQL-like mechanism for the semantic query.

### Knowledge representation in DIR

**Fig. 3:**
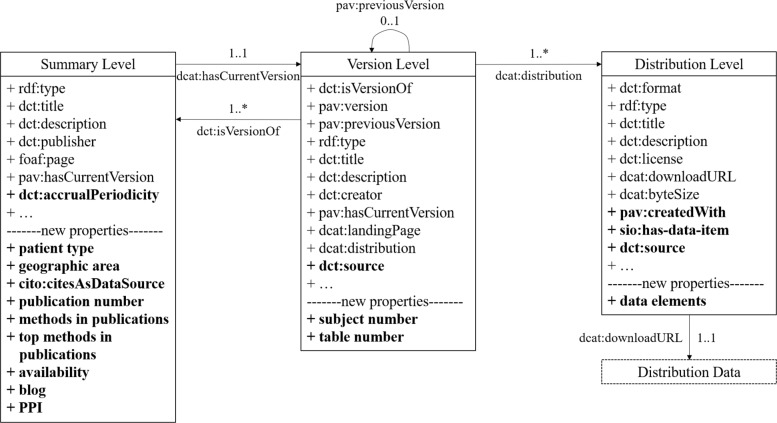
Schema of extended W3C dataset description profile

To represent dataset metadata in a standard manner that is findable, accessible, interoperable, and reusable, we adopt the W3C Dataset Description Profile [9] as the basis of a metadata description model. This profile categorizes dataset metadata in three levels: summary, version, and distribution (see Fig. 3). The summary level is the highest-level description of datasets for the most common information that is independent of specific versions, such as titles, publishers, and homepage links. The version level, as an intermediary of summary and distribution levels, captures version-specific metadata, such as version identifiers and issue dates. The distribution level describes specific forms of a specific version. It includes the most detailed information and guidance, such as data items and links to achieve data. In the DIR prototype, each level of a dataset is a page. Since a dataset can have multiple versions and each version may have various forms, each dataset is described by at least three pages—a summary level (the entrance), at least one version level, and at least one distribution level. For each level, the W3C profile defines a set of suggested data elements, properties, and ranges. The properties that describe datasets are all selected from existing ontologies, such as the Provenance Authoring and Versioning ontology (pav) [17], Data CATalog vocabulary (dcat) [18], and the CItation Typing Ontology (cito) [19]. Since levels depend on each other, several specific properties are defined to link different level pages of a dataset, such as pav:hasCurrentVersion (links the summary level to the version level) and dcat:distribution (links the version level to the distribution level).

The DIR framework further extends the W3C Dataset Description Profile standard to incorporate properties that represent specific knowledge needed to address the learning needs of health informatics novices. Figure 3 presents the extended properties in bold font, and Table 1 illustrates the detailed extension. As shown in Table 1, four major types of knowledge are currently extended: descriptive information, publication-related metadata, detailed data elements, and blogs. Among publication-related metadata, the Publication-based Popularity Index (PPI) is a special property used to compare and rank datasets [20]. Blogs of each dataset are unique and important metadata in the DIR and elaborate on concrete instructions, sample codes, and results that guide an easy start for practice. These blogs targeting novices are written by experienced dataset users, so direct support is strongly provided.

**Table 1:** Extended dataset metadata based on W3C dataset description profile

Property	Original value in W3C profile	Extended value in DIR	Level	Description
**Descriptive information**				
dct:accrualPeriodicity	IRI	IRI or xsd:string	Summary level	Dataset update frequency
Patient type	N/A	xsd:string	Summary Level	Patient type in a dataset (e.g., ICU patients)
Geographic area	N/A	xsd:string	Summary level	Geographic area of a dataset (e.g., city, region, and state)
Availability	N/A	xsd:string	Summary level	Availability of a dataset (e.g., public or proprietary)
Dct:source	IRI	IRI or xsd:string	Version level and distribution level	Data source provenance
Subject number	N/A	xsd:integer	Version level	Number of subjects (e.g., number of patients)
Table number	N/A	xsd:integer	Version level	Number of tables
pav:createdWith	IRI	IRI or xsd:string	Distribution level	Tools used to create a dataset
**Publication-related metadata**				
cito:citesAsDataSource	N/A	IRI	Summary level	Link to publications or a collection of publications using a dataset
Publication number	N/A	xsd:integer	Summary level	Number of publications that analyze a dataset
Methods in publications	N/A	xsd:string	Summary level	Methods used in publications to analyze a dataset
Top methods in publications	N/A	xsd:string	Summary level	Top (usually top 10) methods used in publications to analyze a dataset
PPI	N/A	xsd:float	Summary level	A publication-based popularity index for dataset ranking
**Detailed data elements**				
sio:has-data-item	IRI	IRI or xsd:string	Distribution level	Item listing (e.g., tables and entities)
Data elements	N/A	xsd:string	Distribution level	Data elements (e.g., attributes)
**Blogs**				
Blog	N/A	IRI	Summary level	Links to blogs of a dataset

### Datasets in current DIR prototype

The current implementation of the DIR includes 12 representative datasets in healthcare, of which 3 datasets—Healthcare Cost and Utilization Project (HCUP) [21], Truven Health MarketScan (MarketScan) [22], and Medical Information Mart for Intensive Care (MIMIC) [23]—are retained from the previous DIR version [24]; nine others are selected from working group notes discussed by domain experts at the UNC Charlotte Health Informatics and Outcomes Research Academy [25]. The nine extended datasets are National Health and Nutrition Examination Survey (NHANES) [26], SEER-Medicare Linked Database (SEER-Medicare) [27], National Longitudinal Study of Adolescent to Adult Health (Add Health) [28], Minimum Data Set (MDS) [29], Clinical Practice Research Datalink (CPRD) [30], The Health Improvement Network (THIN) [31], Premier Healthcare Database (Premier) [32], Clinformatics Data Mart (Clinformatics) [33], and Humedica NorthStar (Humedica) [34].

There are several reasons to choose these datasets. To verify the universality of DIR knowledge representation, they cover most types of healthcare datasets, including claims data (SEER-Medicare, CPRD, MarketScan, Premier, and Clinformatics), electronic medical records (MDS and THIN), hospital data (SEER-Medicare, HCUP, MIMIC, and Humedica), laboratory data (Clinformatics), surveys (NHANES and Add Health), and contextual data (Add Health).

Additionally, these datasets are all large and complex datasets in healthcare, of which three (SEER-Medicare, Add Health, and Clinformatics) even include multiple types listed in the prior paragraph. Most of them have a large number of subjects. For example, HCUP includes the largest collection of longitudinal hospital care data in the United States, and MarketScan consists of nearly 240,000,000 patients’ fully integrated, de-identified, individual-level healthcare claims data. In addition to the large amount of data, the diversity of data and the complexity of the structure make novices more difficult to understand and begin to analyze the datasets. For example, MIMIC contains not only numeric and textual data stored in tabular forms, such as lab results and electronic documentation but also graphical data that are stored separately, such as bedside monitor trends and waveforms. Adopting these graphical signals requires not only a deep understanding of data themselves but also sufficient computer skills to convert them into analyzable data and adequate knowledge to decide analytical methods.

Moreover, these datasets are all widely used in healthcare data analytics. A large number of research articles have been published based on these datasets. By searching in PubMed Central (PMC) [35]—an authoritative electronic archive of free full-text biomedical and life sciences journal articles supported by U.S. National Institutes of Health’s National Library of Medicine (NIH/NLM)—the most studied dataset, NHANES, was mentioned in 37,485 articles, while the least discussed dataset among them, Humedica, was mentioned in up to 22 articles. On average, each dataset contributes to more than 4000 publications in PMC.

Finally, these datasets are representative of both public and proprietary datasets. Among the 12 datasets, two of them (NHANES and MIMIC) are public for research purposes, nine of them (SEER-Medicare, HCUP, MDS, CPRD, MarketScan, THIN, Premier, Clinformatics, and Humedica) are proprietary, and one dataset (Add Health) provides both public- and contractual-use data. In novices’ perspectives, complex proprietary datasets are even more challenging than public datasets because they have difficulty retrieving information elsewhere to help them build up a good understanding accurately and quickly.

In the current implementation, we manually extracted most of the metadata from dataset documentations and semi-automatically extracted metadata about analytical methods from publications. The extracted metadata was first stored in the RDF triple format in Excel spreadsheets and imported into MW, using a Python script that converts spreadsheets to MW importable XML files. To ensure the accuracy of manually extracted metadata, a team of health informatics research assistants was formed to review and correct these metadata iteratively.

### Extraction of analytical methods from publications

Data analytical methods that have been successfully applied to datasets are important knowledge for data science learners. To deliver this knowledge, we developed a semi-automatic method to extract various analytical methods that had been used in published articles that analyzed the specific datasets in the DIR. For this task, we first developed an ontology of data analytical methods, Method Ontology (MethodOntology.owl [36]), which extended an existing ontology. Based on the Method Ontology, we developed a rule-based Named Entity Recognition (NER) pipeline to extract instances of analytical methods reported in selected publications.

We used PMC as the data resource and downloaded full-text articles that mentioned the 12 datasets, using the keyword identification method in [20]. In total, 48,282 PDF-format publications were obtained. The publication number of each dataset is shown in Table 2. To preprocess these publications, we developed a pre-processor, written in the Bash command and Python programming language, which included three major steps: (1) converted PDF files to plain text; (2) excluded proceedings and articles that only cited a dataset without analyzing it; and (3) selected relevant content by removing reference sections. After preprocessing, 25,201 publications remained.

**Table 2:** Publication numbers of 12 datasets

Dataset	# of PDF-format articles in PMC	# for method extraction after preprocessing	# that analyzing datasets
NHANES	37,485	16,213	10,674
SEER-Medicare	2569	2276	1627
Add Health	1881	1477	1028
HCUP	1785	1398	993
MDS	1337	1053	584
CPRD	1014	735	477
MarketScan	985	920	614
THIN	733	678	434
MIMIC	237	206	152
Premier	165	158	95
Clinformatics	69	65	49
Humedica	22	22	9
**Total**	**48,282**	**25,201**	**16,736**

**Fig. 4:**
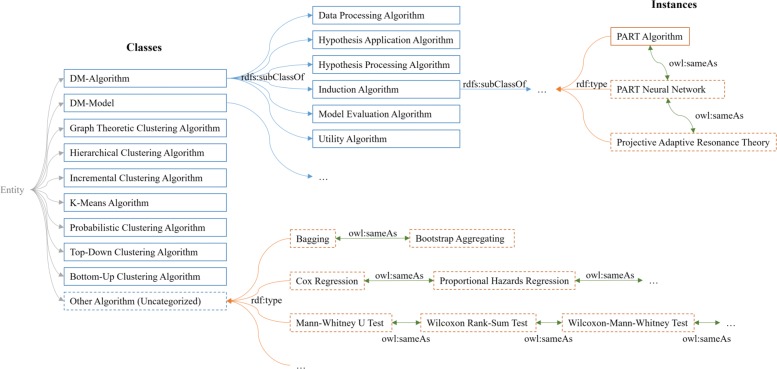
Structure of major method classes and some examples of extended instances in Method Ontology. Extended elements are shown in dashed boxes

The Method Ontology describes data analytical methods, which include all major machine learning, data mining, and statistical methods. This ontology extends the Data Mining Knowledge Base (DMKB.owl) of the Data Mining OPtimization Ontology (DMOP version 5.4), which was originally designed to support informed decision-making in the data mining (DM) process [37]. The DMKB.owl describes instances of DMOP concepts, including individual algorithms in popular data mining software, such as RapidMiner and Weka. For the method extraction purpose, the Method Ontology extended it by adding and linking new methods, which were extracted in a training set of dataset publications, and synonyms of all method instances. Figure 4 shows the structure of major method classes and a few examples of extended instances in the Method Ontology.

**Fig. 5:**
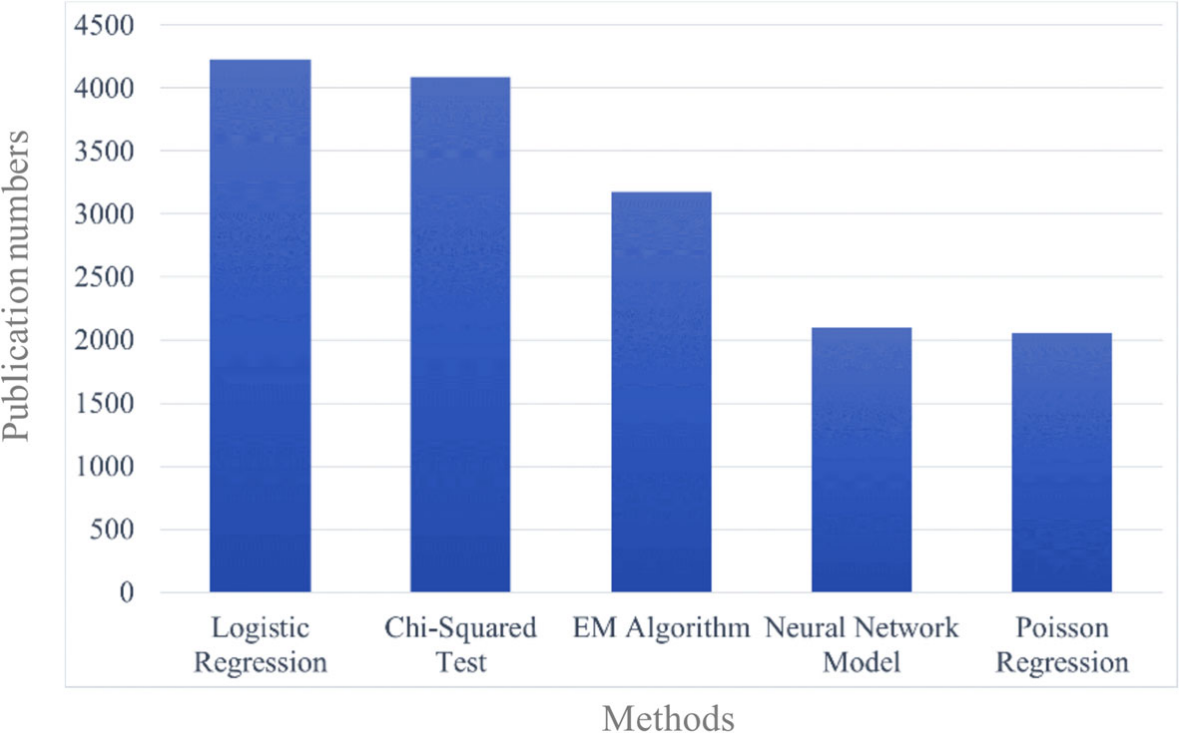
The most frequently used methods in publications of 12 datasets

A rule-based NER was carried out in the Clinical Language Annotation, Modeling, and Processing Toolkit (CLAMP) [38]—a Natural Language Processing (NLP) software—and was handled by a pipeline that included a sentence detector, a tokenizer, and a dictionary lookup component. The input to this pipeline included the preprocessed publications as well as a method dictionary with semantic labels generated from the Method Ontology. After all potential method entities in the publications were extracted, a post-processor was developed to refine these entities and to combine synonyms for further metadata representation. As a result, method entities were extracted and were represented on summary level pages of the datasets. Assume that a publication that analyzed a dataset mentioned at least one analytical method in the full text. In that case, more than half (16,736 out of 25,201) of the preprocessed publications would have analyzed these datasets. According to the publications, the most frequently used methods for the 12 datasets, as well as proportions of publications that utilized the corresponding method, are shown in Table 3. Among these methods, logistic regression, mentioned in 4229 publications, was the most frequently used (see Fig. 5).

**Table 3:** Ten most frequently used methods to analyze each dataset

Dataset	Methods				
NHANES	EM algorithm	Neural network model	Wilcoxon signed-rank test	Poisson regression	Chi-squared test
	29.55%	19.63%	16.69%	15.02%	14.85%
	Kruskal-Wallis test	Logistic regression	Log-rank test	Linear regression	T-test
	14.32%	12.56%	12.17%	10.04%	8.51%
SEER-medicare	Chi-squared test	Logistic regression	Cox regression	Log-rank test	Survival analysis
	54.52%	50.83%	39.64%	17.46%	14.87%
	T-test	Regression model	Kaplan-Meier survival estimates	Linear regression	Propensity score matching
	11.12%	10.45%	9.34%	8.85%	7.01%
Add health	Logistic regression	Chi-squared test	Linear regression	Regression model	Principal component analysis
	50.00%	33.17%	13.13%	9.82%	8.07%
	ANOVA	Poisson regression	T-test	Propensity score matching	Cox regression
	7.49%	5.74%	5.06%	3.40%	3.40%
HCUP	Logistic regression	Chi-squared test	Linear regression	T-test	Regression model
	57.91%	48.44%	20.24%	18.03%	15.61%
	ANOVA	Poisson regression	Cox regression	Mann-Whitney U test	Bootstrap
	9.87%	9.06%	7.45%	7.35%	4.23%
MDS	Logistic regression	Chi-squared test	Linear regression	Regression model	T-test
	42.12%	39.73%	17.29%	14.90%	13.53%
	ANOVA	Cox regression	Mann-Whitney U test	Bootstrap	Survival analysis
	13.18%	9.93%	7.19%	4.11%	3.77%
CPRD	Logistic regression	Cox regression	Chi-squared test	Poisson regression	Propensity score matching
	42.35%	31.03%	18.87%	12.37%	10.48%
	Linear regression	Regression model	Survival analysis	T-test	Kaplan-Meier survival estimates
	9.85%	8.60%	6.08%	5.66%	4.61%
MarketScan	Chi-squared test	Logistic regression	Cox regression	T-test	Poisson regression
	[-2pt]47.88%	43.32%	19.22%	12.87%	12.21%
	Propensity score matching	Linear regression	Regression model	ANOVA	Fisher’s exact test
	10.91%	9.93%	9.77%	6.68%	5.86%
THIN	Logistic regression	Cox regression	Chi-squared test	Poisson regression	Regression model
	37.33%	26.04%	23.27%	12.44%	9.91%
	Inverse probability weighting	Linear regression	T-test	Survival analysis	Propensity score matching
	8.99%	8.53%	8.06%	6.91%	6.68%
MIMIC	Logistic regression	Chi-squared test	T-test	Mann-Whitney U test	Regression model
	45.39%	20.39%	17.76%	15.79%	14.47%
	Support vector machine	Linear regression	Cox regression	Kolmogorov-Smirnov test	K-nearest neighbors
	14.47%	11.84%	11.18%	9.87%	9.21%
Premier	Chi-squared test	K-means	Decision tree model	Logistic regression	Propensity score matching
	41.05%	38.95%	27.37%	21.05%	14.74%
	Kruskal-Wallis test	Linear discriminant analysis	Regression model	Linear regression	T-test
	13.68%	11.58%	11.58%	8.42%	8.42%
Clinformatics	Linear regression	Bootstrap	Regression model	Kruskal-Wallis test	Chi-squared test
	44.90%	28.57%	20.41%	14.29%	12.24%
	F-test	Cox regression	Logistic regression	ANOVA	Survival analysis
	12.24%	10.20%	10.20%	8.16%	6.12%
Humedica	Chi-squared test	Logistic regression	Bootstrap	Fisher’s exact test	Cox regression
	33.33%	22.22%	22.22%	22.22%	11.11%
	T-test	Linear regression	Propensity score matching	Survival analysis	Ensemble learning
	11.11%	11.11%	11.11%	11.11%	11.11%

We evaluated the pre-processor and the method extraction steps separately. The results showed that the 95% confidence interval of the pre-processor’s accuracy was [92.26%, 99.39%], and the precision and recall of the analytical method extraction were 93.82% and 90.53%, respectively.

### Dataset learning and question answering

Once the dataset knowledge is extracted and represented, the direct way to query the knowledge is to write SPARQL-like queries in the semantic search mechanism provided by SMW. While this direct method is powerful, it requires an understanding of the Semantic Web and SPARQL, which is clearly burdensome to novices. Our current approach to addressing this issue is to offer a simplistic question-answering functionality by identifying the most popular questions that novices ask and providing ready-to-use queries. We created a parameterized question page for each representative question, where users can simply input words and click the Run Query button to obtain precise answers. The list of current parameterized question pages is shown in Table 4.

**Table 4:** Eighteen parameterized question pages in current DIR

Data-driven questions	Which datasets include some specific information/data elements?
	Which datasets have more than a specific number of subjects?
Method-driven questions	Which datasets can I apply a specific method to?
Introduction questions	What does a dataset talk about?
	How to get a specific dataset?
	What are the methods that publications used with a specific dataset?
	What are the publications using a specific dataset?
	Is a specific dataset open to the public?
	How many subjects are there in a specific dataset?
	How many tables are there in a specific dataset?
	What are the different tables/files in a database?
	What are the data elements in a specific dataset?
	What are the patient types that a specific dataset handles?
	How frequently are data updated in a dataset?
	How many times is a dataset cited?
	Who reports the data in a specific dataset?
	What is the geographic area of a dataset?
	What is the full name of a dataset?

For example, if users are curious about which datasets can successfully utilize the Support Vector Machine, they can simply visit the “Which datasets can I apply the method to” question page, choose or type in “Support Vector Machine,” and click the Run Query button to obtain “Answer: NHANES, CPRD, THIN, HCUP, MDS, MIMIC.” The dataset result is in order based on the PPI recommendation. In this example, the query below has already been embedded in the question page template:


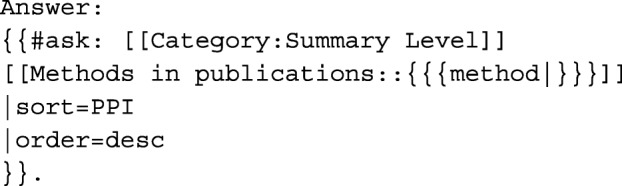


As another example, if users need to investigate large datasets that have more than 1,000,000 subjects, they can refer to the parameterized question page—“Which datasets have more than a specific number of subjects”—that includes the following query:


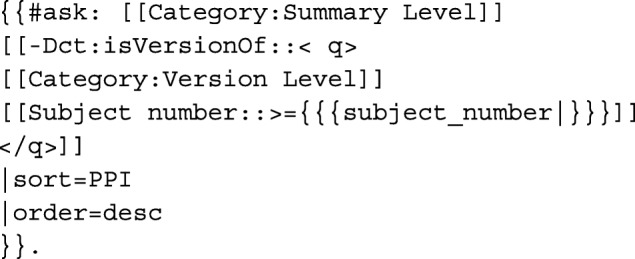


To determine the most popular questions that novices ask, we analyzed a variety of resources, including a publication that guides novices to conduct high-value dataset analysis [39], questions labeled as “dataset” on question-and-answer sites (e.g., Quora [40] and Stack Exchange [41]), and opinions from health informatics novices through interviews.

## Results and discussion

**Fig. 6:**
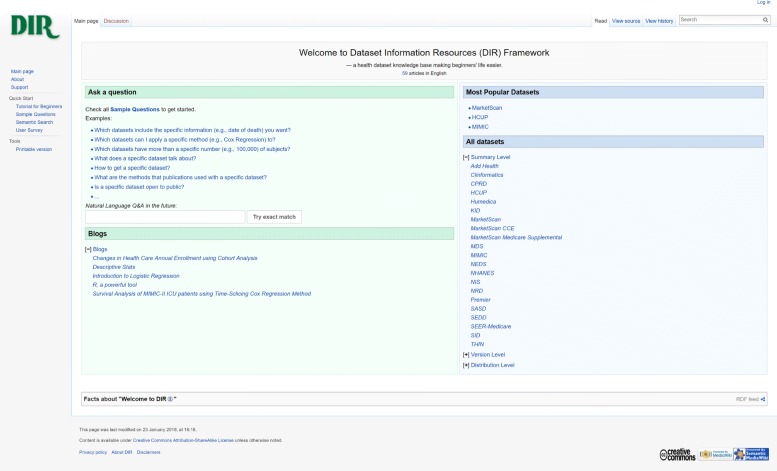
Current DIR homepage

A prototype of the DIR has been developed and released. It is accessible via https://cci-hit.uncc.edu/dir/. The current DIR homepage is shown in Fig. 6. Built on the foundation of the Semantic Web and the extended W3C dataset description profile, we have provided knowledge about 12 representative datasets in healthcare—NHANES, SEER-Medicare, Add Health, HCUP, MDS, CPRD, MarketScan, THIN, MIMIC, Premier, Clinformatics, and Humedica—and five blogs. To facilitate novices’ question answering, 18 ready-to-use questions (Table 4) have been provided. In addition, the more powerful semantic search function is available for users who are familiar with SPARQL. To ease usability, a tutorial and a support page with an issue tracker and a feedback form are also provided.

At the time of this paper’s submission, the DIR prototype contained 264 pages. The average page loading time was 1.44 s. The current approach to add a new dataset includes both manual metadata extractions (from documentations with team review-based quality check) and semi-automatic knowledge extractions (from publications using NLP technologies). To add a new dataset, the current approach takes approximately one day, in general, for manual extractions and a few minutes for semi-automatically extracting analytical methods, excluding the time to collect publications.

We have conducted a survey and collected feedback from 15 target users who were novices in healthcare data research. Of the target users, 40% had a background in health informatics, and 86.7% had a background in data analytics. We asked the subjects to compare Google, DIR, and other resources in seven use cases and also asked for general comments. The survey results indicated that 73.3% of users, on average, preferred the DIR in these use cases. Significantly, 100% of them preferred the DIR in the case of finding datasets that included a particular data element; 93.3% preferred the DIR when they wanted to adopt a specific analytical method; and 86.7% preferred the DIR in the case of discovering large-enough datasets, such as a dataset that had more than 1,000,000 subjects. In terms of more general knowledge, users tended to rely on broader resources. For example, only 60% of users chose the DIR when they were looking for basic descriptions of a dataset or tutorials about gaining access, while others felt more comfortable on searching in Google, browsing the official website, or using both DIR and other resources simultaneously. Overall, the DIR obtained a score of 86.7% in helpfulness, 83.8% in ease of discovering datasets, 82.9% in ease of question answering, and 82.9% in the scale of meeting users’ expectations about healthcare dataset information resources.

According to comments in survey responses, users highlighted the advantages of the DIR as targeted and novice-friendly. As some users commented: “It filters out the irrelevant information and is more structural”; “Beginner-friendly. Information is exhibit[ed] clearly to the user”; and “Sample questions and semantic search are very useful for researchers to find the right dataset or information, or we can say it looks more intelligent than other search engine[s] like [G]oogle.”

However, the DIR clearly has several limitations in this initial phase. (1) The current DIR prototype still relies on manual extractions in part, which is time-consuming and labor-intensive for DIR developers during dataset extending. This limitation has two possible ways to be improved. One refers to the entity linking and typing topic that is intensely discussed in Semantic Web conferences, such as the Open Knowledge Extraction Challenge (OKE) [42] at the European Semantic Web Conference (ESWC). The other way, mentioned by the CEDAR project, involves promoting an authoring-friendly ecosystem in the healthcare dataset community and encouraging researchers to contribute open metadata. (2) Currently, we do not differentiate subclasses of analytical methods, that is, the statistical methods, such as Chi-Square Test, are listed together with machine learning methods, such as Ensemble Learning. Further classification of methods based on the Method Ontology will be needed to address more detailed user questions. (3) As one user commented in the survey: “For now, finding a question is not that hard. However, if the question set becomes larger, then I think it can cause a problem. Somehow you need to facilitate this part,” which reveals that preparing query-embedded question-answering pages can only be a temporary solution. When the system is expanded, a real natural language question-answering functionality should be implemented. Actually, question answering is a stand-alone topic in the Semantic Web community and has been discussed over decades in conferences (e.g., the open challenge on Question Answering over Linked Data (QALD) [43] at ESWC) and publications (e.g., [44–48]). (4) As another user mentioned in the survey: “I’m not sure if researchers will trust the information on DIR.” Rely on simple quality check approaches is one of the limitations. To ensure quality and to gain user trust, a systematic quality assurance method needs to be developed and reported.

## Conclusions

We conclude that it is feasible to develop a DIR that provides value for entry-level health informatics students and researchers. Knowledge about datasets is effectively represented in Semantic Web technologies. At this stage, the DIR has already been able to provide comprehensive and relevant knowledge of 12 important healthcare datasets, which is expected to improve health informatics novices’ ability to learn data analysis using suitable datasets.

In contrast to bioinformatics datasets, of which most data elements have already been represented in RDF at the knowledge level, the DIR will continue focusing on the healthcare datasets that are usually at a lower level granularity.

Further development is underway to improve efficiency, accuracy, and scalability. Suitable directions for expansion include two levels: content and function. The content level adds more healthcare datasets, identifies more types of knowledge for target users, and involves a systematic quality assurance method to ensure the quality of metadata. The function level includes developing a natural language-based question-answering component, more automated methods to extract knowledge, intelligent functionalities to compare similar datasets, and collaborative features, such as discussion forums that allow users to help each other and suggest new content.

## Abbreviations

Add Health: National longitudinal study of adolescent to adult health
bioCADDIE: Biomedical and healthcare data discovery index ecosystem
CCDC: Cambridge crystallographic data centre
CEDAR: Center for expanded data annotation and retrieval
cito: CItation typing ontology
CLAMP: Clinical language annotation, modeling, and processing toolkit
Clinformatics: Clinformatics data mart
CPRD: Clinical practice research datalink
dcat: Data catalog vocabulary
DIR: Dataset information resource
DM: Data mining
DMKB: Data mining knowledge base
DMOP: Data mining optimization ontology
ESWC: European semantic web conference
FAIR: Findable, accessible, interoperable, and re-usable
HCLS: Health care and life sciences
HCUP: Healthcare cost and utilization project
Humedica: Humedica NorthStar
MarketScan: Truven health MarketScan
MDS: Minimum data set
MIMIC: Medical information mart for intensive care
MW: MediaWiki
NCBI: National center for biotechnology information
NER: Named entity recognition
NHANES: National health and nutrition examination survey
NIF: Neuroscience information framework
NIH: National institutes of health
NLM: National library of medicine
NLP: Natural language processing
OKE: Open knowledge extraction challenge
pav: Provenance authoring and versioning ontology
PMC: PubMed central
PPI: Publication-based popularity index
Premier: Premier healthcare database
QALD: Question answering over linked data
RDF: Resource description framework
SEER-Medicare: SEER-medicare linked database
SGIM: Society of general internal medicine
SMW: Semantic MediaWiki
SPARQL: SPARQL protocol and RDF query language
THIN: The health improvement network
WWW: World wide web
